# A Potential Synergy between Incomplete Arsenic Methylation Capacity and Demographic Characteristics on the Risk of Hypertension: Findings from a Cross-Sectional Study in an Arsenic-Endemic Area of Inner Mongolia, China

**DOI:** 10.3390/ijerph120403615

**Published:** 2015-03-31

**Authors:** Yongfang Li, Da Wang, Xin Li, Quanmei Zheng, Guifan Sun

**Affiliations:** Research Center of Environment and Non-Communicable Disease, School of Public Health, China Medical University, Shenyang 110013, China; E-Mails: liyongfang_17@163.com (Y.L.); wangda@mail.cmu.edu.cn (D.W.); lixin@mail.cmu.edu.cn (X.L.); qmzheng@mail.cmu.edu.cn (Q.Z.)

**Keywords:** arsenic, arsenic methylation capacity, hypertension, metabolites, demographic characteristics

## Abstract

Inefficient arsenic methylation capacity has been associated with various health hazards induced by arsenic. In this study, we aimed to explore the interaction effect of lower arsenic methylation capacity with demographic characteristics on hypertension risk. A total of 512 adult participants (126 hypertension subjects and 386 non-hypertension subjects) residing in an arsenic-endemic area in Inner Mongolia, China were included. Urinary levels of inorganic arsenic (iAs), monomethylarsonic acid (MMA), and dimethylarsinic acid (DMA) were measured for all subjects. The percentage of urinary arsenic metabolites (iAs%, MMA%, and DMA%), primary methylation index (PMI) and secondary methylation index (SMI) were calculated to assess arsenic methylation capacity of individuals. Results showed that participants carrying a lower methylation capacity, which is characterized by lower DMA% and SMI, have a higher risk of hypertension compared to their corresponding references after adjusting for multiple confounders. A potential synergy between poor arsenic methylation capacity (higher MMA%, lower DMA% and SMI) and older age or higher BMI were detected. The joint effects of higher MMA% and lower SMI with cigarette smoking also suggest some evidence of synergism. The findings of present study indicated that inefficient arsenic methylation capacity was associated with hypertension and the effect might be enhanced by certain demographic factors.

## 1. Introduction

Arsenic, a hazardous naturally occurring element, is widely distributed in the environment [1]. Humans can be exposed to arsenic through air [2], food [3] or medicinal use [4], but the major route of exposure is consumption of arsenic-contaminated drinking water [5]. It has been estimated that more than 200 million people, including a large number of children, are exposed to high levels of arsenic in drinking water around the world [6]. A recent finding highlights that almost 19.6 million Chinese people are at risk of being affected by chronic arsenic poisoning [7]. Long-term arsenic exposure has been reported to be associated with cancers of skin, lung, bladder, liver and kidney. [8]. Increasing evidence has also suggested a possible role of arsenic in the development of chronic non-cancer diseases [6], among which hypertension is one of the focus of concern.

Hypertension is an important public health problem due to its high frequency and concomitant risks of cardiovascular and kidney disease [9]. It has been identified as the dominating risk factor for mortality, and is ranked third as a cause of disability-adjusted life-years [9,10]. The association between increased prevalence of hypertension and the long-term arsenic exposure via drinking water was first reported among persons living in the arsenic-endemic areas of Taiwan in 1995 [11]. The positive association was subsequently demonstrated by researches from Bangladesh [12,13] and United States [14], and the effect was revealed to be in a dose-response manner [15]. A recent systematic review including 11 cross-sectional studies that summarized by Abhyankar *et al.* identifies the positive association between elevated arsenic exposure in drinking water and the prevalence of hypertension, but suggests that the implications of this association from a causal viewpoint are still unclear especially under lower arsenic exposure conditions [16]. Given to the widespread arsenic exposure via drinking water, even a small association between arsenic exposure and hypertension might have an extensive impact on morbidity and mortality, and therefore it is important to further explore the association from multiple perspectives. Up to now, most of reports have discussed the association from the perspective of arsenic concentrations in the drinking water. Studies focus on the influence of arsenic metabolism on the susceptibility of hypertension are limited.

In drinking water, arsenic is usually found in the form of inorganic arsenate or arsenite. The ingested inorganic arsenic (iAs) is primarily metabolized in liver through methylation processes. Though the exact pathway of arsenic methylation is controversial [17,18], iAs is well-recognized to be methylated in two steps, first to monomethylarsonic acid (MMA) and then to dimethylarsinic acid (DMA). This methylation facilitates the excretion of iAs from the body because the end products, MMA and DMA, are more water soluble and more readily excreted in the urine [19]. Epidemiological studies have shown that iAs methylation is incomplete in humans, and the composition of urinary arsenic metabolites varies from person to person, even when populations exposed to the same level of arsenic in drinking water [20]. The relative proportion of the urinary arsenic species as well as two methylation indices, the primary methylation index (PMI) and the secondary methylation index (SMI), have been used to reflect the methylation capacity of exposed individuals in several studies [21,22,23].

Previously, the methylation metabolism of arsenic was considered as a detoxification process. However, studies in recent years indicate that intermediate metabolites of arsenic methylation, especially trivalent MMA, have stronger cytotoxicity and genotoxicity than iAs itself [24,25]. The new understanding of arsenic metabolism raises an interesting challenge in evaluating the relationship between arsenic-induced adverse health effects and different methylation capacity. Previous findings have shown that subjects who present a lower capacity of arsenic methylation, characterized as a higher proportion of urinary MMA or a lower SMI, are more susceptible to arsenic-caused diseases, including peripheral vascular disease [26], skin lesions [22] and cancers of skin [27], bladder [28] and breast [29]. In our previous research in Shanxi Province of China, we also detected a positive relationship between high levels of urinary MMA and the increased susceptibility of arsenic-related hypertension [30]. More importantly, the associations between poor arsenic methylation capacity and arsenic-induced diseases have been found can be modified by other potential modifiers [31,32,33,34]. Results from a study in Argentina have shown that the association between urinary MMA% and the risk of bladder cancer is stronger among smokers than nonsmokers [32]. A more recent finding from our group also indicates that the lower urinary DMA% and SMI may act synergistically with older age, lower BMI and male gender on arsenicosis risk [35]. Based on our previous findings and other reports, it is possible to hypothesize that joint effects may be exist between lower arsenic methylation capacity and potential modifiers in relation to the risk of arsenic-induced hypertension. To test the hypothesis, therefore, we performed a cross-sectional research among populations residing in a known arsenic-endemic area of Inner Mongolia, China. The objective of our study was to explore the interactions of arsenic methylation capacity with demographic characteristics on the hypertension risk. We supposed that the findings of our work might be able to provide a new point to understanding the relationship between arsenic exposure via drinking water and the susceptibility of hypertension, and also present some public health strategies to reduce this disease burden in arsenic-exposed populations.

## 2. Materials and Methods

### 2.1. Study Population

Our study was performed in Tuoketuo County of Inner Mongolia, which is one of the most serious arsenic endemic regions located in northwest China. Patients with chronic arsenicosis caused by consumption of drinking water containing high levels of naturally occurring arsenic in the region were firstly identified in 1990s. Populations living in this region solely rely on groundwater for drinking and domestic use. According to the report of local public health government, the arsenic concentration in groundwater ranged from < 0 to 760 µg/L. Although a water improvement project has been gradually conducted in this area, there are still some villages using unimproved groundwater. The subjects of this study were recruited from the villages whose water supply had not yet been improved by the government so as to ensure a clear arsenic exposure history of each participant.

Given to the variation of arsenic metabolism among different ethnic groups, only residents of Han nationality were recruited into the study. Additionally, adult residents (above 18 years old) who lived in the study county more than 10 years were considered as eligible subjects. Those participants who had consumed seafood or arsenic-containing traditional Chinese medicines (e.g., Niu Huang Jie Du Pian, a known Chinese patent medicine) in the past seven days before study were excluded. To eliminate the confounding effect of hormones on arsenic metabolism, pregnant and breastfeeding women were also ruled out in our study. Subjects who routinely used prescription medications known to interfere with one-carbon metabolism (e.g., methotrexate and antiepileptic drugs) and with severe diseases (e.g., tumors) were also excluded. Finally, 512 subjects of the total 816 residents who voluntarily participated in the study met the criteria mentioned above and were confirmed as study subjects. Informed consent was read and signed by all subjects before administrating a detailed interview. Information about demographic characteristics, socioeconomic status, lifestyle of cigarette smoking and alcohol consumption, dietary habits, type of work, medical history, family members and education conditions were obtained by well-trained interviewers based on a structured questionnaire. Trained doctors conducted comprehensive physical examinations. The measurement of standing height and body weight was completed according to the standard methods. Body mass index (BMI) was computed as weight in kilograms divided by height in square meters. This study was conducted according to the Declaration of Helsinki Ethical Principles for Medical Research Involving Human Subjects and was approved by Ethics Committee of China Medical University.

### 2.2. Blood Pressure Measurement and Diagnosis of Hypertension

In the current study, blood pressure was measured three times with a mercury sphygmomanometer in sitting position after rest and relaxation for at least 15 min based on the standard protocol recommended by the World Health Organization [36]. Systolic blood pressure and diastolic blood pressure were defined at the first and fifth Korotkoff sounds, respectively. The lowest measured value was viewed as the suitable value and was used for final analysis. Subjects who were found with high blood pressure were rechecked for another two measurements a few days later to confirm the finding of hypertension (once per visit). Hypertension was defined in our study as a systolic blood pressure ≥ 140 mmHg, or a diastolic blood pressure ≥ 90 mmHg and/or a history of hypertension under regular treatment with antihypertension medications.

### 2.3. Arsenic Exposure Measurement

Because the concentration of arsenic in each tube well was determined and reported to the family by the local public health government, it was considered as reliable in the present study. Cumulative arsenic exposure (CAE) was calculated by the arsenic concentrations in the tube wells which is the subjects used in their residential duration multiplied by the duration of water consumption (milligrams per liter × years). The CAE was calculated for all subjects.

### 2.4. Biochemical Analysis

Before physical examination, all subjects were asked to fast for at least 12 h. Fasting venous blood sample was drawn from each participant. An aliquot blood sample was used immediately for the determination of blood glucose concentration by HemoCue Glucose 201RT glucometer (HemoCue, Lake Forest, CA, USA). To evaluate the liver function, serum concentrations of aspartate transaminase (AST) and alanine transaminase (ALT) were then analyzed in laboratory by using an autoanalyzer (Hitachi 7170A; Hitachi; Ltd., Tokyo, Japan) with reagents obtained from Nanjing Jiancheng Bioengineering Institute (Nanjing, China).

### 2.5. Measurement of Urinary Arsenic Metabolites

The mid-stream of the first morning void urine (15 mL) was collected from all participants in polypropylene tubes and kept in ice box. Exactly 1 mL of urine was kept separately for creatinine (Cr) determination. All urine samples were then immediately transferred to Centre for Disease Control and Prevention of Hohhot, Inner Mongolia, and stored at −20 °C. All samples were then shipped with dry ice to the Laboratory of Arsenic Analysis in China Medical University (Shenyang, China) by air and stored at −80 °C, and finally measured for urinary arsenic metabolites within 3 months.

The determination of urinary arsenic metabolites, iAs, MMA and DMA, was performed by atomic absorption spectrophotometer (AA-6800, Shimadzu Co., Kyoto, Japan) with an arsenic speciation pretreatment system (ASA-2sp, Shimadzu Co.). Arsenic speciation analysis was based on the well-established hydride generation of volatile arsines, followed by cryogenic separation in liquid nitrogen. The detection limits of the hydride generation and atomic absorption spectrometry (HG-AAS) method for the three arsenic species (iAs, MMA and DMA) were 1 ng, respectively. In our previous researches, the detailed process of the method has been described [21,30,37]. Briefly, 1 mL of urine that has been stored at −80 °C was thawed at room temperature, and was digested with 2 N-NaOH solutions at 100 °C for 3 h in a 15-mL polymethylpentene test tube followed by dilution with mili-Q water. The assay samples were stirred every 1 h. The absorbance of arsenic in the digested urine samples was determined at a wavelength of 193.7 nm. A Standard Reference Material of freeze-dried urine (SRM 2670, National Institute of Standards and Technology (NIST), Gaithersburg, MD, USA), which contain 480 ± 100 μg/L arsenic was used as a quality control to check the validity of urinary arsenic species measurement. The values determined in our laboratory were 474 ± 20 μg/L. The reliability of arsenic species separation was evaluated by the analytical recoveries of added arsenic species. Spiking urine samples with 10 μg/L of iAs, MMA and DMA resulted in recoveries of 81%–92%, 88%–98% and 89%–103% for iAs, MMA and DMA, respectively. Urinary Cr, used to account for urine dilution in spot urine samples, was determined by Jaffe reaction with a commercial kit (Jiancheng Biological Institute of Nanjing).

### 2.6. Statistical Analysis

Total arsenic (tAs) concentration in the urine was calculated by summing the concentrations of iAs, MMA and DMA. Concentrations of three arsenic metabolites (iAs, MMA and DMA) and tAs were adjusted by individual urinary Cr level to correct for urine dilution. According to the measurement of urinary arsenic species, the proportions of urinary arsenic metabolites (iAs%, MMA% and DMA%) and two methylation indices, the PMI and SMI were calculated to reflect the arsenic methylation capacity. The PMI and SMI were calculated as (MMA + DMA)/tAs and DMA/(MMA + DMA), respectively. Since the positively skewed distribution of the urinary arsenic metabolites, natural logarithmic transformations were used to normalize their distributions and the geometric means as well as the 95% confidence interval (CI) were determined. Student *t* test was then used to assess the differences of continuous variables among different groups, and the Chi-square test was used to explore the differences of category variables. The univariate and multivariate logistic regression analyses were used to evaluate the urinary arsenic methylation indices and the risk of hypertension. In the multivariate model, we simultaneously adjusted for multiple confounders including sex (male/female), age (continuous), BMI (continuous), alcohol consumption (yes/no), cigarette smoking (yes/no) and CAE (continuous). The results of logistic analyses were presented as odd ratio (OR) along with their 95% CIs.

To evaluate potential biological interactions between arsenic methylation indices and demographic characteristics on the risk of arsenic-related hypertension, we calculated the relative excess risk due to additive interaction (RERI) and corresponding CIs [38]. Only demographic characteristics revealed to be risk factors of hypertension by the univariate logistic regression were included in the biological interaction analyses. The RERI and its 95% CI were calculated according to the standard delta method [39]. The RERI > 0 indicates the presence of synergistic effect, and a 95% CI that is positive and excludes zero corresponds to *p* < 0.05 for RERI. These calculations were performed according to Andersson *et al.* [40], using an Excel spreadsheet (EpiNET 2008). We used *p* value < 0.05 for statistical significance. All statistical analyses were performed using SPSS software version 16.0 (SPSS Inc., Chicago, IL, USA).

## 3. Results

### 3.1. Characteristics of the Study Population

A total of 512 subjects, including 126 hypertension subjects and 386 non-hypertension subjects were included in our study. The baseline characteristics of all participants by status of hypertension are showed in Table 1. For categorical variables, the proportion of cigarette smokers in subjects with hypertension was significantly higher than those without, but the proportions of male subjects and alcohol consumer were not significantly different.

**Table 1:** Baseline characteristics of hypertension and non-hypertension subjects from arsenic-endemic area in Inner Mongolia, China (*n* = 512).

Characteristics	Hypertension (*n* = 126)	Non-Hypertension (*n* = 386)	*p*-Value
*Category variables ^a^*			
Male	51 (40.48%)	153 (39.64%)	0.867
Cigarette smoker	42 (33.33%)	93 (24.09%)	0.041 ^*^
Alcohol consumer	25 (19.84%)	74 (19.17%)	0.869
*Continuous variables ^b^*			
Age (years)	65.17 ± 12.19	53.60 ± 14.38	<0.001 ^**^
BMI (kg/m^2^)	24.20 ± 4.15	22.18 ± 3.17	<0.001 ^**^
CAE (mg/L-year)	0.61 ± 0.80	0.38 ± 0.83	0.007 ^**^
Cr (mg/L)	758.43 ± 494.96	844.13 ± 501.48	0.095 ^&^
FBG (mmol/L)	5.58 ± 1.36	5.25 ± 1.07	0.013 ^*^
AST (U/L)	33.17 ± 9.71	32.35 ± 12.73	0.537
ALT (U/L)	21.39 ± 10.88	20.99 ± 10.38	0.730
** *Urinary arsenic levels ^c^*			
iAs (µg/g Cr)	11.75 (9.77, 14.13)	7.94 (6.91, 8.91)	0.008 ^**^
MMA (µg/g Cr)	20.42 (17.38, 23.99)	13.18 (12.02, 14.45)	<0.001 ^**^
DMA (µg/g Cr)	99.72 (87.10, 114.82)	79.43 (74.13, 87.10)	0.012 ^*^
tAs (µg/g Cr)	138.04 (117.49, 158.49)	104.71 (97.72, 114.82)	0.002 ^**^
*Arsenic methylation indices ^c^*			
iAs%	8.51 (7.59, 9.55)	7.59 (6.92, 8.13)	0.128
MMA%	14.79 (13.80, 15.85)	12.59 (12.02, 13.01)	<0.001 ^**^
DMA%	72.44 (70.79, 74.13)	76.16 (75.28, 77.04)	0.003 ^**^
PMI	0.90 (0.89, 0.91)	0.89 (0.87, 0.91)	0.357
SMI	0.82 (0.80, 0.84)	0.85 (0.84, 0.85)	<0.001 ^**^

Notes: *Abbreviates*: BMI, body mass index; CAE, cumulative arsenic exposure; Cr, creatinine; FBG, fasting blood glucose; AST: aspartate transaminase; ALT: alanine transaminase; iAs, inorganic arsenic; MMA, monomethylarsonic acid; DMA, dimethylarsinic acid; tAs, total arsenic; PMI, primary methylation index; SMI, secondary methylation index. ^a^ Values are number (%). ^b^ Values are mean ± SD. ^c^ Values are geometric mean (95% confidence interval). ^&^ *p* < 0.10; ^*^ *p* < 0.05; ^**^ *p* < 0.01.

For the continuous variables, age, BMI, CAE, and fasting blood glucose (FBG) were significantly higher among hypertension subjects than those non-hypertension subjects, while no differences of urinary Cr, AST and ALT were detected between the two groups. With respect to urinary arsenic metabolites, statistically significant higher levels of iAs, MMA, DMA and tAs were found in case group than non-case group. Subjects with hypertension also possessed a higher urinary MMA%, lower urinary DMA% and SMI compared with subjects without hypertension. However, no differences concerning iAs% and PMI were found between the two groups.

### 3.2. Association between Urinary Arsenic Methylation Indices and Hypertension Risk

Table 2 lists the results of multiple logistic regression analysis for hypertension in relation to tertiles of urinary arsenic methylation indices. Subjects with MMA% in the upper tertiles carried a higher risk of hypertension while compared to subjects with MMA% in the lowest tertiles (OR = 1.81, 95% CI: 1.10, 2.99). After adjustment for confounding factors, the risk of hypertension in the upper tertiles of urinary MMA% was not statistically higher than that in the lowest tertiles (OR = 1.70, 95% CI: 0.93, 3.11; *p* = 0.084). Participants with the highest urinary DMA% showed a significant lower risk of hypertension as compared to their reference tertiles after adjustment for confounding factors (OR = 0.37, 95% CI: 0.20, 0.68). In comparison to subjects with the lowest tertiles of SMI, a significant decreasing risk of hypertension was detected in those with the highest (before adjustment: OR = 0.53, 95% CI: 0.33, 0.88; after adjustment OR = 0.47, 95% CI: 0.26, 0.86) and middle tertiles of SMI (before adjustment: OR = 0.61, 95% CI: 0.38, 1.00; after adjustment OR = 0.44, 95% CI: 0.25, 0.79) both before and after adjustment for multiple confounding factors, respectively. The findings indicated a negative relationship between SMI and the risk of hypertension. No associations with regard to iAs% and PMI and the risk of hypertension were found in the current study populations.

**Table 2:** Odd ratios (95% CIs) for association of arsenic methylation indices with the risk of hypertension among subjects from arsenic-endemic area in Inner Mongolia, China (*n* = 512).

Characteristics ^a^	Hypertension (*n* = 126)	Non-hypertension (*n* = 386)	OR (95% CI)	*p*-Value	OR (95% CI) ^b^	*p*-Value
iAs%						
<6.70	41	129	1.00		1.00	
6.70–10.63	47	124	1.19 (0.73, 1.94)	0.478	1.64 (0.93, 2.91)	0.089 ^&^
>10.63	38	133	0.90 (0.54, 1.49)	0.678	1.34 (0.75, 2.40)	0.370
MMA%						
<11.52	33	137	1.00		1.00	
11.52–15.56	41	130	1.31 (0.78, 2.20)	0.307	1.03 (0.57, 1.86)	0.934
>15.56	52	119	1.81 (1.10, 2.99)	0.020 ^*^	1.70 (0.93, 3.11)	0.084 ^&^
DMA%						
<73.40	48	122	1.00		1.00	
73.40–80.83	44	127	0.88 (0.55, 1.42)	0.603	0.65 (0.37, 1.13)	0.128
>80.83	34	137	0.63 (0.38, 1.04)	0.072 ^&^	0.37 (0.20, 0.68)	0.001 ^**^
PMI						
<0.90	38	133	1.00		1.00	
0.90–0.94	47	124	1.33 (0.81, 2.17)	0.261	1.20 (0.68, 2.09)	0.531
>0.94	41	129	1.11 (0.67, 1.84)	0.678	0.74 (0.42, 1.32)	0.331
SMI						
<0.83	54	116	1.00		1.00	
0.83–0.87	38	133	0.61 (0.38, 1.00)	0.048 ^*^	0.44 (0.25, 0.79)	0.006 ^**^
>0.87	34	137	0.53 (0.33, 0.88)	0.013 ^*^	0.47 (0.26, 0.86)	0.014 ^*^

Notes: *Abbreviations*: iAs, inorganic arsenic; MMA, monomethylarsonic acid; DMA, dimethylarsinic acid; PMI, primary methylation index; SMI, secondary methylation index. ^a^ Cut points were determined by tertiles of overall study participants. ^b^ Adjusted by sex, age, body mass index, cigarette smoking, alcohol consumption and cumulative arsenic exposure. ^&^ *p* < 0.10; ^*^ *p* < 0.05; ^**^ *p* < 0.01.

### 3.3. Joint Effects of Urinary Arsenic Methylation Capacity with Potential Modified Variables on Hypertension Risk

The joint effects of urinary MMA% and DMA% with age or BMI on hypertension risk are showed in Table 3. Compared to younger participants with lower MMA%, older subjects had higher urinary MMA% suggested a higher risk of hypertension (OR = 8.64, 95% CI: 4.22, 17.72) than older persons carrying a lower urinary MMA% (OR = 6.52, 95% CI: 3.18, 13.37) as well as those who were younger and had a higher urinary MMA% (OR = 2.12, 95% CI: 0.96, 4.67). In accordance with MMA%, the OR for hypertension in populations characterized by lower DMA% and older age (OR = 6.07, 95% CI: 3.05, 12.07) was greater than those with higher DMA% and older age (OR = 4.47, 95% CI: 2.31, 8.62) as well as younger subjects carrying lower DMA% (OR = 0.98, 95% CI: 0.45, 2.15). The calculated RERI for the interaction between higher MMA% or lower DMA% and older age was 1.00 (95% CI: −3.43, 5.43) and 1.62 (95% CI: −1.54, 4.78), respectively.

**Table 3:** The joint effects of urinary arsenic methylation indices with age and BMI on hypertension risk among subjects from arsenic-endemic area in Inner Mongolia, China (*n* = 512).

Characteristics	Joint Effect between Urinary Arsenic Methylation Indices and Age				Joint Effect between Urinary Arsenic Methylation Indices and BMI			
	Age	Cases/Non-Cases (*n*)	OR (95% CI) ^a^	RERI (95% CI)	BMI	Cases/Non-Cases (*n*)	OR(95%CI)^ b^	RERI (95%CI)
MMA% ^c^								
≤13.41	≤57	14/137	1.00		≤22.21	14/100	1.00	
>13.41	≤57	18/102	2.12 (0.96, 4.67)		≤22.21	26/116	1.77 (0.81, 3.81)	
≤13.41	>57	40/65	6.52 (3.18, 13.37) ^**^		>22.21	40/102	4.16 (1.95, 8.84) ^**^	
>13.41	>57	54/82	8.64 (4.22, 17.72) ^**^	1.00 (−3.43, 5.43)	>22.21	46/68	5.09 (2.41, 10.76) ^**^	0.17 (−2.68, 3.02)
DMA% ^c^								
>77.61	≤57	18/118	1.00		≤22.21	18/91	1.00	
≤77.61	≤57	14/121	0.98 (0.45, 2.15)		≤22.21	22/125	1.35 (0.63, 2.87)	
>77.61	>57	46/74	4.47 (2.31, 8.62) ^**^		>22.21	46/101	3.42 (1.69, 6.90) ^*^	
≤77.61	>57	48/73	6.07 (3.05, 12.07) ^**^	1.62 (−1.54, 4.78)	>22.21	40/69	4.48 (2.18, 9.19) ^**^	0.71 (−2.36, 3.78)
SMI ^c^								
>0.85	≤57	15/134	1.00		≤22.21	14/101	1.00	
≤0.85	≤57	17/105	1.69 (0.88, 3.69)		≤22.21	26/115	2.32 (1.06, 5.07) ^*^	
>0.85	>57	37/70	5.08 (2.51, 10.28) ^**^		>22.21	38/103	4.67 (2.08, 9.60) ^*^	
≤0.85	>57	57/77	8.59 (4.26, 17.32) ^**^	2.81 (−1.35, 7.04)	>22.21	48/67	6.36 (3.00, 13.50) ^**^	0.57 (−2.81, 3.95)

Notes: *Abbreviates*: MMA, monomethylarsonic acid; DMA, dimethylarsinic acid; SMI, secondary methylation index; BMI, body mass index. ^a^ Adjusted by sex, body mass index, cigarette smoking, alcohol consumption and cumulative arsenic exposure. ^b^ Adjusted by sex, age, cigarette smoking, alcohol consumption and cumulative arsenic exposure. ^c^ Cut point was determined by median value of each variable. ^*^ *p* < 0.05, ^**^ *p* < 0.01.

With respect to the interaction effect of MMA% with BMI, participants with higher MMA% and higher BMI had a 5.09-fold increased risk of hypertension (OR = 5.09, 95% CI : 2.41, 10.76) compared to participants with lower MMA% and lower BMI. The RERI between higher MMA% and higher BMI was 0.17 (95% CI: −2.68, 3.02), which indicated a similar synergistic effect between MMA% and BMI on the risk of hypertension. Similarly, the effect size of lower DMA% and higher BMI on the risk of hypertension was evident stronger (OR = 4.48, 95% CI: 2.18, 9.19) than their corresponding references (higher DMA% and lower BMI). The calculated RERI for the interaction of lower DMA% with higher BMI was 0.71 (95% CI: −2.36, 3.78).

The interaction effects between SMI and age or BMI in relation to hypertension risk are also reported in Table 3. After the joint effects between SMI and age were combined, participants whose SMI values lower than 0.85 and whose age higher than 57 had a 8.59-fold (OR = 8.59, 95% CI: 4.26, 17.32) increased risk of hypertension in comparison to participants whose SMI higher than 0.85 and whose age lower than 57. However, the joint effect of lower SMI with older age was not statistically significant greater than the sum of their individual effects, with the RERI being 2.81 (95% CI: −1.35, 7.04). The risk of hypertension for subjects carrying lower SMI and higher BMI was significantly higher than those with higher SMI and lower BMI (OR = 6.36, 95% CI: 3.00, 13.50). RERI estimates indicated that the joint effect of lower SMI with higher BMI did not statistically exceed additivity (RERI = 0.57, 95% CI: −2.81, 3.95).

Table 4 shows the effects of the interaction between urinary MMA%, DMA% or SMI and cigarette smoking status. The joint effects of higher MMA% or lower SMI with cigarette smoking on hypertension risk were significantly greater than their corresponding references. The RERI for synergistic effect was 0.35 (95% CI: −1.86, 2.56) between higher MMA% with cigarette smoking and was 0.70 (95% CI: −1.71, 3.11) between lower SMI with cigarette smoking. No apparent synergism effect was found between lower DMA% and cigarette smoking, with an RERI being −0.81 (95% CI: −3.24, 1.62).

**Table 4:** The joint effects of urinary arsenic methylation indices with cigarette smoking on hypertension risk among subjects from arsenic-endemic area in Inner Mongolia, China (*n* = 512).

Characteristics	Variables	Hypertension Cases/Subgroup (*n*)	OR (95% CI) ^a^	RERI (95% CI)
MMA% ^b^	Cigarette smoking			
≤13.41	Never	43/168	1.00	
>13.41	Never	41/125	1.43 (0.81, 2.53)	
≤13.41	Ever	11/34	2.12 (0.83, 5.42)	
>13.41	Ever	31/59	2.91 (1.37, 6.15) ^**^	0.35 (−1.86, 2.56)
DMA% ^b^	Cigarette smoking			
>77.61	Never	47/161	1.00	
≤77.61	Never	17/31	2.90 (1.23, 6.83) ^*^	
>77.61	Ever	37/132	1.60 (0.90, 2.84)	
≤77.61	Ever	25/62	2.69 (1.24, 5.85) ^*^	−0.81 (−3.24, 1.62)
SMI ^b^	Cigarette smoking			
>0.85	Never	42/169	1.00	
≤0.85	Never	42/124	1.72 (0.97, 3.04)	
>0.85	Ever	10/35	2.04 (0.78, 5.31)	
≤0.85	Ever	32/58	3.45 (1.63, 7.33) ^**^	0.70 (−1.71, 3.11)

Notes: *Abbreviates*: MMA, monomethylarsonic acid; DMA, dimethylarsinic acid; SMI, secondary methylation index. ^a^ Adjusted by sex, age, body mass index, alcohol consumption and cumulative arsenic exposure. ^b^ Cut point was determined by median value of each variable. ^*^ *p* < 0.05, ^**^ *p* < 0.01.

## 4. Discussion

In the study, we evaluated interactions of arsenic methylation capacity with demographic characteristics on the risk of hypertension among populations exposed to arsenic via drinking water in Inner Mongolia, China. We found the joint effects of a lower methylation capacity (higher MMA%, lower DMA% and SMI) with older age or higher BMI or cigarette smoking on hypertension risk were probably additive. Although the RERI estimates in our study were imprecise, the findings might also indicate a possible synergistic effect between a lower methylation capacity and potential modifiable factors.

From the angle of public health, iAs from drinking water is the primary source of human exposure. After ingestion, the iAs mainly metabolized in the liver to generate trivalent and pentavalent methylated arsenicals. Recent studies have found that the trivalent forms of arsenical metabolites are more toxic than their pentavalent analogs and even more than iAs itself, with MMA ^III^ being the most toxic arsenic metabolite [24,25]. This new understanding of arsenic metabolism has questioned the traditional belief that methylation was a detoxification pathway of iAs, and also raised an interesting challenge in evaluating the relationship between arsenic-induced adverse health effects and different methylation capacity. Findings from Steinmaus *et al.*, [41] have shown that subjects with the upper tertiles of urinary MMA% have higher risk of lung cancer compared to those with the lowest tertiles. In the article, meanwhile, the authors reviewed previous researches regarding to the association between urinary MMA% or MMA/DMA ratios and other arsenic-related diseases, including cancers of skin and bladder, skin lesions, peripheral vascular disease, atherosclerosis and hypertension. The odd ratios for arsenic-related diseases in every study except for one are higher in those with higher MMA% or higher MMA/DMA ratios. The consistent results in these researches provide a fairly large body of evidence linking the MMA% with the arsenic-related disease risks. Additionally, more and more recent findings and our previous researches have indicated that other inefficient arsenic methylation indices, including a lower percentage of DMA and a lower SMI in the urine were positively related to breast cancer, development delay and skin lesions [22,29,42]. Likewise, in the current study, we examined the association between arsenic methylation capacity and hypertension risk. Our data showed that subjects with hypertension had a higher percentage of urinary MMA, lower urinary DMA and SMI than those without (Table 1), which was similar with a previous research performed by Huang *et al.* in Taiwan [23]. Furthermore, a significant higher risk of hypertension was observed for participants carrying the lowest tertiles of DMA% and SMI as compared to their corresponding references after adjusting for multiple confounders (Table 2). A borderline higher risk of hypertension was also suggested in subjects with the highest tertiles of MMA% when compared to those with the lowest tertiles of MMA% in the multivariate logistic regression model (Table 2). However, no association was obtained for iAs% and PMI with the risk of hypertension (Table 2). Collectively, these findings indicate a positive relationship between inefficient arsenic methylation capacity and the risk of hypertension, and also suggest a more decisive role of the second methylation step, which is defined by low fraction of more toxic MMA and high fraction of less toxic DMA in the urine, than the first step in arsenic methylation. These results were quite compatible with a prospective study which has shown that subjects with poor arsenic methylation capacity (high MMA%, low DMA% and SMI) having an increased risk of developing cardiovascular diseases than those with efficient metabolism [34].

The exact underlying mechanisms for the positive associations between arsenic and hypertension or other cardiovascular diseases have not been fully understood. Previous studies have found that arsenic exposure through drinking water can induce oxidative stress, endothelial injury and smooth muscle cell proliferation [43]. Soucy *et al.* [44] further demonstrated that chronic exposure to environmentally relevant concentrations of arsenic could promote pathological neovascularization and time-dependent expressions of cardiovascular genes which related to neovascularization and tissue remodeling in mice. Meanwhile, researches focus on the role of MMA ^III^ on the mechanisms of arsenic-induced cardiovascular toxicological effects have found that MMA ^III^ can induce smooth muscle dysfunction [45], potentiate the agonist-induced vasoconstriction [46] and disrupt bioactivity of endothelial nitric oxide synthase [47]. These vascular toxic effects are suggested can in turn lead to dysregulation of normal physiological hemodynamics and aberrant blood pressure changes. Taken together, all of these researches provide evidence for the role of arsenic as well as the arsenic metabolism in arsenic-induced cardiovascular diseases, including hypertension.

Although data from several studies have suggested the positive association between arsenic methylation capacity and diverse health hazards, high inter-individual variability and differential arsenic susceptibility imply that it is necessary to assess not only the indices of arsenic methylation capacity, but also the synergistic effect of arsenic methylation capacity with potential modifiers on increasing arsenic-related diseases burden. Previous findings have indicated that the arsenic methylation process can be influenced by various factors, including genetic polymorphisms, demographic characteristics (e.g., sex, age, and cigarette smoking) and nutritional factors (e.g., dietary folate, vitamin B12 and B6) [48,49]. The potential synergism effects of incomplete arsenic methylation capacity with older age and cigarette smoking regarding to the risk of cardiovascular diseases have been reported in a prospective study from Bangladesh [34]. A similar synergy between chewing tobacco and arsenic metabolism in women for the risk of skin lesions was also observed by Lindberg *et al.* [33]. In this study, we evaluated the interaction of the inefficient arsenic methylation capacity with demographic characteristics concerning to the hypertension risk. According to the results of univariate logistic regression, only age, BMI and cigarette smoking were revealed to be risk factors of hypertension among our population. Therefore, the combined effects analyses were focused on the interactions of arsenic methylation indices with age, BMI as well as the smoking status on the risk of hypertension. We found that the joint effects of higher MMA% with older age, higher BMI and cigarette smoking were greater than the sum of their separate effects, respectively (Table 3 and Table 4). For example, the calculated RERI for the interaction between MMA% and older age was 1.00, indicating that for every unit increase in urinary MMA% and age, the odd ratio was 1.00 more than if there was no interaction. With respect to DMA%, the RERI estimates indicated that lower DMA% and older age or higher BMI might act in a potential synergistic manner to increase the risk of hypertension (Table 3). Analysis of older age, higher BMI and cigarette smoking also produced some evidence of a synergistic interaction with lower SMI (Table 3 and Table 4). Taken together, the findings in our study suggest a possible synergistic effect between a lower methylation capacity and some demographic characteristics on the hypertension risk.

Several epidemiological studies have demonstrated that age is one of factors affect arsenic metabolism [21,26,48]. Our previous findings in Inner Mongolia have suggested that children possess higher capacity for secondary methylation of arsenic than adults when exposed to the same concentrations of iAs in drinking water [21]. For adults only, study from Taiwan also indicates that the levels of MMA% increased while the DMA% and SMI decreased along with the age increasing [26]. The aging effect on arsenic metabolism may be because it partly reflects the length of residence, which is an indicator of duration of arsenic exposure. Additionally, the aging process can probably be associated with a variety of functional changes in the organs involved in metabolism or retention of the metabolites of arsenic [48]. BMI, as an indicator of overall nutritional status, also has been shown positively associated with arsenic methylation capacity [50,51]. Although the underlying mechanisms for the association is not clear, some evidence suggest that obese and non-obese people may have different composition of gastrointestinal microorganisms [52], which have been shown can methylate some inorganic arsenic to methylated metabolites before systemic metabolism [53,54]. The relationship between cigarette smoking and arsenic metabolism has been extensively studied in many researches, and most of findings support smoking is associated with a poor arsenic methylation capacity [26,33]. The potential mechanisms for this association have not been fully elucidated, but inhibition of enzymes (e.g., arsenic 3-methyltransferase) involved in arsenic methylation and impairment of one-carbon metabolism are suggested as the general causes [33,48]. In light of the findings on potential synergies between BMI or cigarette smoking and lower arsenic methylation capacity observed in our study, smoking cessation and nutritionally adequate diet may have public health significances in prevention of arsenic-induced hypertension, particularly among old people residing in arsenic-endemic areas.

In interpreting our findings, some limitations should be considered. First, due to the large-scale water improvement projects progressively carried out, inhabits exposed to high levels of arsenic in drinking water become increasingly rare. And thus, the number of subjects accorded with our inclusion criteria and finally involved in the analysis was limited. The large variation of CIs around the effect estimates and the statistically insignificant RERI estimates showed in the Table 3 and Table 4 were probably, at least partly, associated with the small sample size in some strata. Second, the participants in our study were quite older (mean age for hypertension subjects: 65.17 ± 12.19 years; mean age for non-hypertension subjects: 53.60 ± 14.38 years). It is possible that the “aging process” can lead to various functional and nutritional changes in the organs that involved in arsenic metabolism, and therefore our study population might be more sensitive to arsenic toxicity, including hypertension. Accordingly, cautions should be exercised in extrapolating our results to other arsenic exposed populations. Third, we are unable to distinguish and quantify MMA ^III^ and MMA ^V^ separately in the urine because of the limitation of analysis system in our laboratory. Final, except for age, BMI and cigarette smoking, other factors including gene polymorphisms (e.g., arsenic 3-methyltransferase; GSTO1 and GSTO2 of glutathione *S*-transferase family) and nutrient intake (e.g., folate, vitamin B12 and vitamin B6) are also reported to be able to affect the arsenic methylation capacity and thus may influence the susceptibility to arsenic-induced hypertension. The potential confounding effects of these factors on the association between poor arsenic methylation capacity and demographic characteristics might be another possible reason for the insignificant RERI estimates that obtained in our study. Further researches focus on the synergy between arsenic methylation capacity and much more potential influencing factors on the risk of hypertension are needed.

## 5. Conclusions

Collectively, in this study, we observed a positive association of incomplete arsenic methylation capacity, which is characterized as lower urinary DMA% and SMI, with the risk of hypertension. Possible synergistic effects of inefficient arsenic methylation capacity with older age, higher BMI and cigarette smoking were also suggested in our data, although the estimates were not precise. These findings highlight that arsenic methylation capacity is a susceptible factor for arsenic-induced hypertension and, more importantly, the association might be enhanced by some demographic characteristics. Future researches with large sample size and considering more potential modifiers are worthy to be carried out to confirm our findings.
